# Maternal health agency in women with a low socioeconomic status: a qualitative study

**DOI:** 10.1080/17482631.2024.2367844

**Published:** 2024-06-24

**Authors:** Sharissa Mandy Smith, Leonieke Willemien Kranenburg, Djanifa da Conceicao, Mijke Pietertje Lambregtse-van den Berg, Régine Patricia Maria Steegers-Theunissen, Hafez Ismaili M’hamdi

**Affiliations:** a Department of Obstetrics and Gynaecology, Erasmus University Medical Centre, Rotterdam, The Netherlands; b Department of Psychiatry, Erasmus University Medical Centre, Rotterdam, the Netherlands; c Midwifery Practice Verlosmoeder, Rotterdam, The Netherlands; d Department of Child and Adolescent Psychiatry/Psychology, Erasmus University Medical Centre, Rotterdam, the Netherlands; e Department of Medical Ethics, Philosophy and History of Medicine, Leiden University Medical Centre, Rotterdam, The Netherlands; f Department of Health, Ethics & Society, Maastricht University, Maastricht, the Netherlands

**Keywords:** Health agency, socioeconomic factors, healthcare uptake, lifestyle, ethics

## Abstract

**Background:**

Health agency refers to one’s capacity to form health-related goals, experience control, and possess the means to pursue them. Low socioeconomic status (SES) is linked to impaired health agency and increased risk of adverse pregnancy outcomes, potentially due to a reduced tendency to seek care. Better healthcare availability may not improve their pregnancy outcomes, and therefore improved understanding of maternal health agency is paramount.

**Methods:**

Semi-structured interviews were conducted with 15 participants who either had children or desired to have them. Low SES was determined by neighborhood median income and educational attainment. A thematic content analyses was conducted.

**Results:**

Two themes emerged: 1) Origin and development of personal goals, and 2) Awareness and competence. Participant’s goals stemmed from cultural norms, personal narratives, and intuition. Integrated goals were those participants valued highly, were aware of, and strived for. Four subthemes were identified in goal-awareness and competence. Internal conflict due to discrepancies between goals and behavior resulted in the need to balance the burdens and benefits of behavior change.

**Conclusion:**

Maternal health agency is a modifiable outcome dependent on goal-awareness and various factors. Impaired agency seemed to stem from lack of goal-awareness rather than an inability to meet established pillars.

## Background

In general, countries that have high-quality and a (partially) subsidized healthcare system offer their citizens a reasonable opportunity to seek medical care. The availability of high-quality care however, does not guarantee its use. That is, the need for healthcare is not necessarily reflected in its uptake. The overall uptake is dependent on multiple factors such as availability, accessibility and affordability of healthcare, as well as the perceived need and demand for healthcare (Peters et al., 2008). The uptake of healthcare is in a sense the product of the right balance between the adequate availability and accessibility of *the offered* care on the one hand, and the adequate experience of the need for care, i.e., the adequate *demand* for care on the other. Challenges in the “demand” side of care have been described in terms of low or impaired health literacy, the experience of insufficient control over one’s health, and the lacking of material and immaterial freedoms one has (i.e., having sufficient time, resources and opportunities to seek care) (Cobb-Clark et al., 2014; Ismaili M’hamdi & de Beaufort, 2021; Lee et al., 2021; Tschaftary et al., 2018).

There are relatively few studies available that pursue the understanding of the sum of factors that can impede the health-seeking behaviours of persons who would benefit from seeking care. A noteworthy exception are studies that centre on “agency” (Grand-Guillaume-Perrenoud et al., 2022; Ismaili M’hamdi & de Beaufort, 2021; Vizheh et al., 2023). Agency refers to the ability to form preferences one has reason to value, make independent and informed decisions, and taking an active role in managing one’s health (Ismaili M’hamdi & de Beaufort, 2021; Naik et al., 2009). This includes health literacy, which entails having the skills and knowledge to understand and use health information. In other words, adequate health agency refers to the ability to know what you want and the capability to take practical steps to reach this goal. Inadequate, or poor health agency refers to either the inability to become aware of your own preference and/or goal, or the inability to act accordingly.

**Figure 1.**
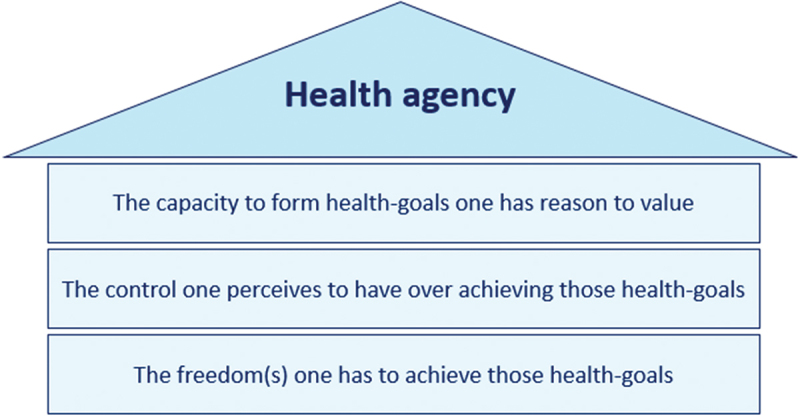
The conceptualisation of health agency as presented by Ismaili M’hamdi (Ismaili M’hamdi & de Beaufort, 2021).

Ismaili M’hamdi and de Beaufort have published an article in which they conceptualize health agency in the form of three pillars (Ismaili M’hamdi & de Beaufort, 2021): 1. The capacity to form health-goals one has reason to value, 2. The control one perceives to have over achieving these health-goals and 3. The freedoms and means (i.e., income, time, motivation etc.) one has to achieve these health-goals (Figure 1). This conceptualization, however, requires further empirical research to assess whether and to which extent impaired maternal health agency in the Netherlands is a relevant factor that leads to a suboptimal uptake of preconception care (PCC) and maternal healthcare. This qualitative study is a first step to achieve this aim.

PCC encompasses professional support in preparing for pregnancy through improving lifestyle, nutrition and health prior to conception, and is known for having a low demand and suboptimal uptake, despite the clear need for PCC (M’hamdi et al., 2018; Maas, Poels, de Kievit, et al., 2022; Poels et al., 2016; Smith et al., 2022). This need is most clear in women with a low SES, who are more at risk of adverse pregnancy outcomes such as preterm birth and even perinatal mortality (de Graaf et al., 2013; Vos et al., 2014; Waelput et al., 2017). The excess risk of perinatal mortality between deprived and non-deprived neighbourhoods builds up to 21% (de Graaf et al., 2013). These increased adverse pregnancy outcomes do not only affect maternal and perinatal health, but might impact lifelong health for the next generations as described by the “Developmental Origins of Health and Disease” (DOHaD) paradigm (Gluckman et al., 2016). The disparities in pregnancy-related outcomes, especially those associated with differences in socioeconomic status (SES), have been qualified as inequities and reducing these disparities is of paramount importance (Ismaili M’hamdi & de Beaufort, 2021).

Understanding the adverse impact of impaired health agency on the challenges of increasing PCC uptake and ameliorating pregnancy-related inequity, may help to better understand the underpinnings of these challenges, and aid in designing interventions and formulating policy which benefits parents-to-be and their offspring. First however, we must explore the concept of pregnancy and child-related health agency (i.e., maternal health agency) in this population and strive to discover which factors may be of influence. This will help determine if the conceptualization of health agency is suitable to better understand the low uptake of PCC in women with a low SES, and help formulate effective policies addressing this challenge.

The aim of this study is to explore the concept of maternal health agency prior to, during and after pregnancy, and discover which factors are of influence in women with a low SES. The secondary aim is to explore if the conceptualization of health agency as presented in Figure 1 is suitable to better understand the low uptake of PCC in this population. In order to reach these aims, we have conducted semi-structured interviews with fifteen women who are at risk for adverse pregnancy outcomes based on their SES.

## Methods

### Study population

We have recruited women through social media posts and an online survey between June 2021 and September 2021, using a purposive sampling method. To promote applications, the survey introduction contained a short explanation of the study, including that participants (i.e., women who were selected and interviewed) would receive compensation for their time investment in the form of a €30 (≈$36) voucher for an online general retail shop. To prevent false applications, the inclusion criteria were not revealed.

The online survey included questions on age, ZIP code, educational level, (previous) pregnancies, and the wish to have a child in the future. A total of 369 women filled in the online survey which was taken down two weeks after the last application. To research maternal health agency, we selected adult women of fertile age (18–45) with children or the wish to have a child in the future, to ensure that participants’ experiences would be (relatively) recent. Further inclusion criteria were having a low to intermediate educational level and living in a neighbourhood with a low median to middle median household income, which served as a proxy for low SES (Galobardes et al., 2006).

**Figure 2.**
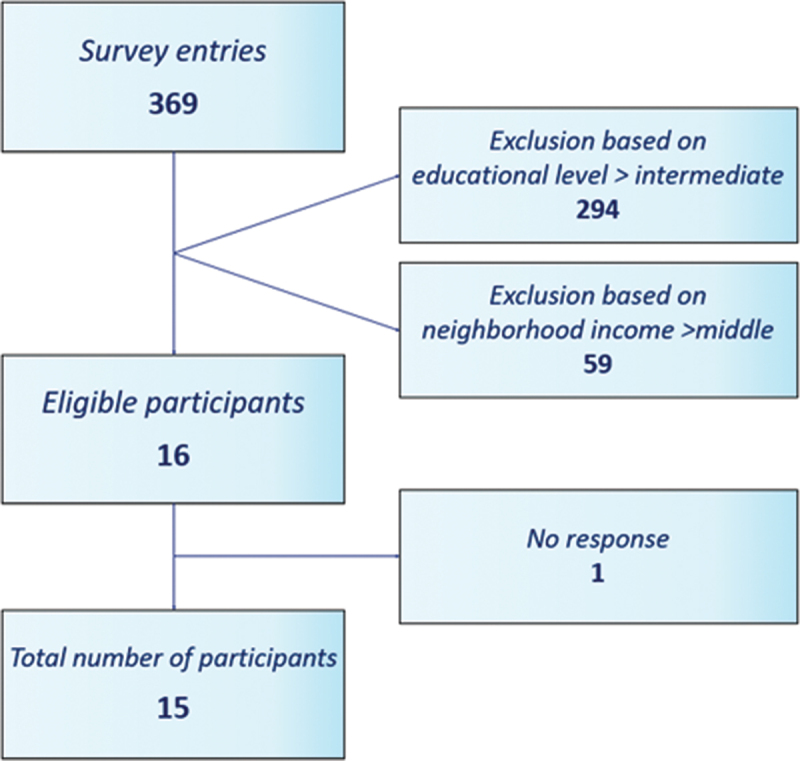
Inclusion flowchart.

Based on these criteria, 16 women were selected for an interview of which 15 were interviewed (Figure 2). Participant characteristics can be found in Table I and Appendix A.

**Table I.** Participants’ characteristics.

Demographics	Participants
	ƞ = 15
**Age in years**	
*Mean (range)*	*28 (18–38)*
*Median (Q1-Q3)*	*28 (24–32)*
**Parity ɳ**	
*Nulliparous, not pregnant*	*5*
*Nulliparous, pregnant*	*1*
*≥1*	*9*
**Educational level† ɳ**	
*Low*	*1*
*Intermediate*	*14*
**Neighbourhood income‡ ɳ**	
*Median= >low - <middle (20–40)*	*14*
*Median= < middle (00–40)*	*1*

^†^Educational level (Luijkx & de Heus, 2008). The Dutch educational levels are subdivided as follows; Low: prevocational education, selective secondary education or lower. Intermediate: vocational education. High: bachelor’s degree, master’s degree or higher.

^‡^The median annual household income of a neighbourhood is determined by the distribution of household income of all households in the country (AlleCijfers.nl [All Numbers], 2021). Low: €15.900 - €21.000 ($18.800–$24.800), Middle: €21.000 - €26.800 ($24.800 - $31.700), Middle-High: €26.800 - €34.600 ($31.700 - $40.900), High >€34.600 (>$31.700).

### Data collection

We have opted for semi-structured interviews to research maternal health agency because this method is suitable to explore participants’ experiences, feelings and views, as well as provide necessary nuance and focus. Additionally, discussing views and choices can be sensitive, and the adaptability of semi-structured interviews allow the researcher to build rapport, helping participants to feel comfortable whilst discussing these topics. Ethical approval from the Erasmus Medical Centre Medical Ethics Committee was obtained (MEC-2021–0465) as well as participants’ informed consent.

The topic list was composed by the first author SS who brainstormed on how best to explore maternal health agency and the factors that influence it. The first draft was then discussed with second author LK and last author HI who are experts in, respectively, psychology and ethics. The adapted topic list was then reviewed by the rest of the team until consensus was reached regarding its content. All interviews were conducted by SS in July 2021 and were carried out using the before mentioned topic list (Appendix B). The topic list was based on the before mentioned conceptualization of health agency (Figure 1). Data saturation was reached after 13 interviews, and was confirmed by the team after conduction of the last two interviews.

### Data analysis

**Figure 3.**
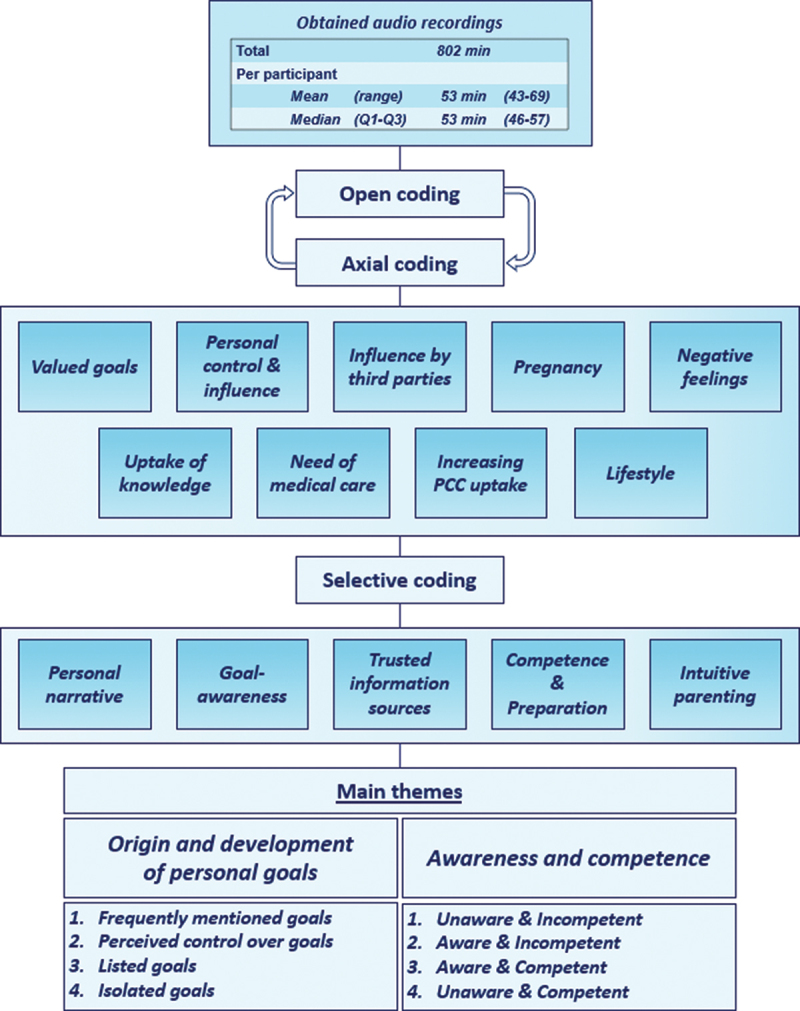
Thematic content analyses: Raw data to themes and subthemes.

All interviews were audiotaped and transcribed verbatim by a professional transcription bureau. The transcripts were then uploaded into NVivo11 software for Windows10 for coding and analysation. The full analysation was performed in Dutch, as to optimize understanding of nuances, connections and distinctions in the data. SS read and annotated all transcripts. The data were analysed using a thematic content analysis, as displayed in Figure 3 (Braun & Clarke, 2006). The first five interviews were open coded, meaning that all possibly meaningful text units and sentences were labelled by SS and discussed with LK.

Subsequently, axial coding of these interviews took place by writing out the open codes on paper and grouping and regrouping them into categories and subcategories, continuously checking if the labelled statements were indeed similar in nature and topic. This led to formation of a tree-structured coding scheme which was checked and adjusted in multiple meetings with LK. SS then proceeded to code the following interviews, simultaneously fitting new codes in the coding scheme, thus adjusting the scheme where necessary. SS, LK and HI reviewed the scheme together by checking the allocated codes and their placement in the coding scheme, implementing further adjustments to make the coding scheme more concise and fitting. This was followed by a meeting attended by SS (medical doctor), LK (psychologist), DdC (midwife), ML (psychiatrist) and HI (ethicist), in which the coding scheme was reviewed, adjusted and subsequently validated. In this same meeting, first steps towards selective coding were taken by discussing which topics emerged as main themes within the data, and identifying the relationships between them. SS continued selective coding by further investigating the main themes and subthemes.

**Figure 4.**
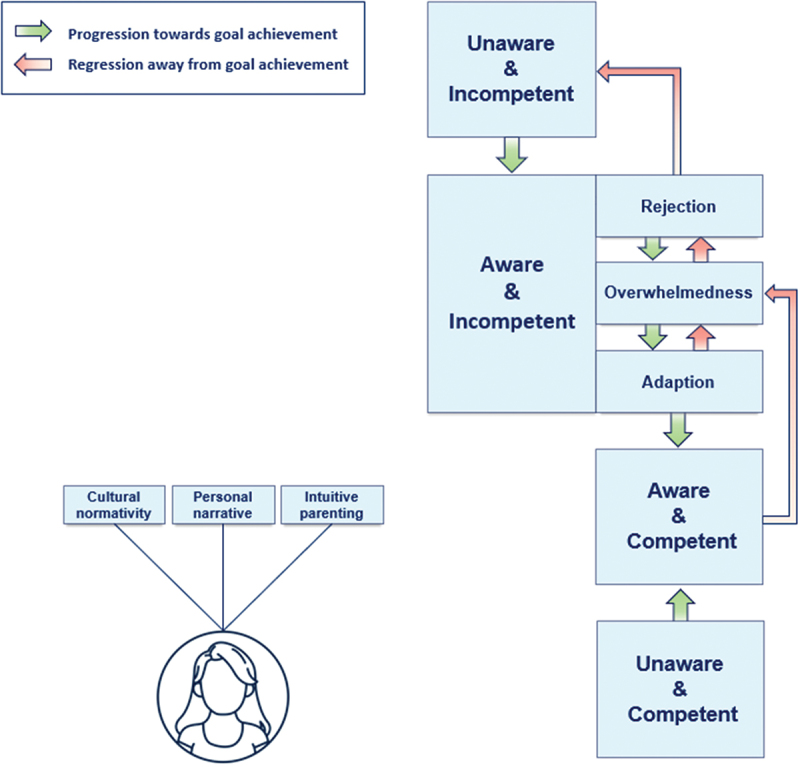
Visual presentation of the findings.

Thereafter, another meeting was set up in which the findings were discussed with the same authors that validated the coding scheme. In this meeting, agreement was reached on the main themes and subthemes, including their interrelationships. LK, DdC, ML and HI reviewed Figure 4 by re-analysing 3 to 4 interviews each, checking for inaccuracies, inconsistencies and missing relevant data. Changes were processed by SS who updated the figure accordingly (Figure 4). After this process was finalized, SS translated the quotes from Dutch to English, verbatim where possible. Subsequently, the translations were checked by 4 female native English speakers, residing in Great Britain, Canada and the United States of America.

## Results

### Main themes

Two main themes emerged from the data: *Origin and development of personal goals* and *Awareness and competence*. Below, these main themes and their respective subthemes will be presented through illustrative quotes from the interviews.

### Main theme 1. The origin and development of personal goals

All participants expressed goals relating to becoming pregnant and parenting their (future) child(dren). Some participants responded immediately, while others took a moment to think about their goals, and possibly form them, before they responded. Noticeable were statements made about specific, practical goals such as limited screen time and forbidding carbonated drinks. Specific goals were usually quickly followed by the underlying reason which originated from the participant’s own experiences or those of family members and friends.

#### 1.1 Frequently mentioned goals

Participants were questioned about goals for their (future) child(ren) prior to (and if applicable during) pregnancy. The most frequently expressed goals included a healthy diet, having friends, performing well in school, and being healthy and happy.

Participant I, 20–25 y.o., mother of 1, 1 miscarriage

I want my child to be strong and have a healthy lifestyle. And having friends growing up, you know? Mainly, I hope that our children will be happy in the home we made for them. And when they are older, that they’ll look back on a happy childhood.

Participant L, 35–40 y.o., mother of 2, 2 miscarriages

I want them to play sports … Not continuously sit at home. And they can always be creative, because I’m creative as well. Study-wise … I want her to do the best she can, but I’m not trying to push her. Being happy is my highest priority for a child. And I’m always honest, always try to be as honest as possible. Health-wise, yeah, being conscious about it. She needs to eat fruit. I’ve put her on a health-oriented school as well.

Participants with children reported that their goals mostly arose after finding out they were pregnant. Further inquiry revealed however, that these goals were already present but were not deemed relevant prior to pregnancy. Otherwise stated, participants’ goals became more defined, and they became more focused on them. For all participants, awareness of their goals was often the result of their own experiences or events they witnessed in others.

Participant J, 20–25 y.o., mother of 2

Thinking about specific goals kind of grew on me throughout my pregnancy. I fantasized about how it would be to have a child before, but nothing concrete. The moment I knew there was a tiny baby in my body, it triggered me to think about how I would take care of it, and start planning.

Participant B, 25–30 y.o., currently pregnant

My only goal is for my child to be healthy. This goal arose at the very beginning: the moment I found out I was pregnant. My sister-in-law had twins and one of them was born with a heart condition. That’s something that takes a hold on you. I immediately thought; What if something like that happens to us.

#### 1.2 Perceived control over goals

Participants discussed their sense of control regarding becoming pregnant and having a healthy child, as well as their parenting goals. Overall, the starting point of this “maternal control” was believed to be from conception onwards. However, four participants believed that maternal influence on infant health starts prior to conception.

Participant G, 20–25 y.o., mother of 1

believe that your influence on a child’s health starts from the moment you become pregnant and after birth. Well no, actually even before you become pregnant. The awareness does strengthen however, after a positive pregnancy test. I used to smoke, and that’s the part you can act on if you want your child to be healthy and happy. This was something I could influence, so I quit before I tried to become pregnant.

Participants stated to feel at least partially in control regarding achieving their goals, but they worried about the influence of external factors. Furthermore, it was frequently commented that, although health can be promoted through healthy behaviour, it is dependent on more than behavioural choices.

Participant D, 25–30 y.o., mother of 5

I do feel that how they develop in school is kind out outside my control and that’s hard for me. Children can be such bullies and I’ve had to deal with that with my oldest. I went and spoke to the teacher and it got resolved, but I did really feel helpless. You want to protect them, but you can’t.

Participant E, 30–35 y.o., mother of 1

I try my best, but I don’t always eat healthy. Sometimes things are just not inside of your control. In pregnancy, of course you try your best, but you can’t fix everything.

Participant K, 30-35 y.o., stepmother to 1

“I don’t necessarily think we’d have trouble achieving our goals, but… I would say I’m … I’m kind of anxious of our world, the outside world. You can try to protect, but yeah, everyone makes mistakes and then you just have to be there for your child”.

When participants were asked what they hoped to achieve through striving for their goals, they mentioned that practical goals such as a healthy diet and having good social skills would hopefully be supportive of a child’s health and happiness in later life. Formulated differently, practical and controllable sub goals are formed to obtain control over a greater main goal.

Participant M, 30–35 y.o.

What I aim to reach with my goals [confident, socially adept, healthy, free, balanced] … I want to say happiness? But that’s a very broad term. I think, happiness and peace. I want my child to be confident in life because that will bring happiness and peace. Not being worried about who you are, if you’re worthy. That’s the first thing that pops up.

#### 1.3 Listed goals

A distinction was noticeable between “listed goals” that were mentioned in a summarized manner, and goals that were mentioned solitary. The listed goals for children mostly included healthy nutrition, having friends and the child being participant’s main priority in life. Listed goals were usually not accompanied by elaboration on why participants valued these goals in particular.

Participant E, 30–35 y.o., mother of 1

When I think about my child’s health and wellbeing, I immediately think about healthy food, exercise, outdoor air and stable relationships. So yeah, adequate food, activity and a healthy environment.

Participant I, 20–25 y.o., mother of 1, 1 miscarriage

I think it’s very important as well that my children have a healthy lifestyle. Oh and I hope we’ll have our own house soon so we can make it a true home. And friends, I really hope they will find friends, growing up.

#### 1.4 Personal goals

Some goals were mentioned solitary, that is to say, they were not mentioned in a checklist like manner. Awareness of these solitary goals seemed to be the result of specific situations or experiences, as they were quickly followed by elaboration on how this goal arose.

Solitary goals seemed to be *valued personally* by the participants, as opposed to merely being *valued in general* by society, making them *personal goals*. When a participant attributed *high personal value* to a goal, the goal seemed to become *“their own”*, or “*integrated in their life”*, planning or actively striving to achieve it. Usually, personal goals originated from participants’ personal narrative: their own experiences, witnessed experiences of others, social and living environment, and personal characteristics and preferences. Another origin of personal goals was found in “*maternal feelings*” or parental intuition; “feeling/knowing what to do” instinctively.

In some interviews, goals that were initially mentioned in a listed manner, were later on mentioned solitary as well, followed by elaboration on the goal’s origin.

Participant A, 20–25 y.o.

I want to be in a mentally healthy place when I get pregnant. If I wasn’t mentally fit, I would definitely postpone getting pregnant. My mother was very depressed when she was pregnant with my youngest brother and it had a tremendous impact on all of us.

Participant B, 25–30 y.o., currently pregnant

My sister-in-law had twins. Whenever they cry, she just gives them her phone. And her eight year old only wants designer clothes. She’s so spoiled. I definitely want to prevent that for our daughter. I want her to be kind, play with normal toys, and be happy.

Participant H, <20 y.o., mother of 1, pregnant, 5 miscarriages

Respect is huge to us as parents, but we want to maintain a friendship. That’s what I had with my mother. She was 19 when she had me and we grew up as friends. It was a nice experience to have a mother so understanding of me because she was so close in age. That’s a goal I definitely want to reach for my children as well; I’m not only your mom, I’m also your friend.

Participant H, <20 y.o., mother of 1, pregnant, 5 miscarriages

“When my son was born, he was tiny and cold. That’s when I said; “He needs to gain weight”. It was instinct, wanting to do everything to protect him.

”Participant O, 35-40 y.o., 1 abortion, 1 miscarriage

“You know? When I got pregnant, I just noticed things changed. I was changing … And the people around me too. It’s a natural process”. [choosing healthier behaviour when you’re pregnant]

## Main theme 2. Awareness and competence

The participants offered insights on how and when they became (more) aware of their goals and their current level of competence regarding achieving the goals they wish to pursue.

“*Goal-awareness*” refers one’s level of awareness regarding a goal they might wish to achieve, if the goal (or its possibility) were known to them. Awareness of a goal was often incited or strengthened by an event or experience. “*Level of competence*” refers to one’s current ability to achieve a goal they wish to pursue. Because “a capability is only as good as its execution” (Bandura, 1982), competence in this study refers to actually striving to achieve the goal, instead of merely possessing the skill to do so.

In the section below, we describe the gradual process of how participants became (more) aware of certain goals and how they progressed from incompetence to competence, and (in some cases) what caused them to regress back to (partial) incompetence.

It is paramount to note that, in the context of this study, (in)competence is not considered a judgement of moral character, but rather an evaluation of participants’ current ability to achieve a goal they wish to strive for.

### 2.1Unaware & incompetent

This section illustrates how unawareness of a goal can lead to behaviour that is not (fully) aligned with the goal a participant wants to achieve. It is important to note that unawareness also entails being unaware of the *realistic possibility* to achieve a certain goal, meaning that a person may be unaware of the fact that they can perform actions that are supportive of achieving their goal, and may instead view achievement as a matter of luck or something they cannot influence.

Participant B, 25–30 y.o., currently pregnant

I didn’t know that you can influence your baby’s health prior to getting pregnant and I didn’t know that being overweight could cause pregnancy diabetes. I wish I knew this before and lost weight before I became pregnant.

Participant E, 30–35 y.o., mother of 1

When I was pregnant, I did mention my eating disorder to the midwife, but I didn’t talk about it with my gynaecologist during our fertility treatment, although I was worried about it. If they would have asked about it, I would have taken the opportunity to discuss my worries.

*Participant I, 20–25 y.o., mother of 1, 1 miscarriage*

After I had that miscarriage, it took a while to get pregnant again. I wondered why and kind of panicked. I started googling and googling, and I found that your partner’s lifestyle habits are important as well. So, the first time, we didn’t quit smoking, but the second time we did. If we would’ve read that information sooner, we would’ve quit prior to getting pregnant the first time.

### 2.2Aware & incompetent

This section illustrates three types of reactions that were seen in response to goal-awareness and incompetence: 1. Rejection, 2. Overwhelmedness and 3. Adaption. (Figure 4)

#### Rejection

Participant O displays awareness regarding the link between smoking marihuana and not getting pregnant. She immediately follows up with a statement that discounts what she said, reducing the importance of quitting.

Participant O, 35–40 y.o., 1 abortion, 1 miscarriage

When I got pregnant, I had quit smoking marihuana for three years. Looking back, I think I might not have gotten pregnant before because I was smoking. I don’t know for sure, didn’t read anything about it … It’s just a feeling. But yeah, I also know women who got pregnant and kept on smoking marihuana even when they were pregnant, so…

#### Overwhelmedness

Participant L displays awareness of her goal to quit smoking for her baby and describes how feeling overwhelmed prompted her to shortly revert back to unaligned behaviour.

Participant L, 35–40 y.o., mother of 2, 2 miscarriages

I quit smoking when I was pregnant, but one time, I had a big fight with the father and then I smoked again because I was so stressed out. He got so pissed off! And then I didn’t really smoke again during my pregnancy.

#### Adaption

Participant K and B display two types of adaption: 1. adaption of behaviour (e.g., changing lifestyle habits), and 2. adaption of the goal (e.g., opting for smoking reduction over cessation).

Participant K, 30–35 y.o., stepmother to 1

After I saw a gynaecologist [because I did not get pregnant], I changed my habits. I started a diet and exercised more. Yeah.

Participant B, 25-30y.o., currently pregnant

“I am a smoker. I have always wanted to quit smoking and I wish I could quit completely, but yeah, I didn’t manage to. I do smoke a lot less than I did before I was pregnant!

### 2.3Aware & competent

Participants who were aware and competent with regard to a specific goal, spoke about this goal in a confident, matter of fact manner. They provided insights on their motivation and showed signs of planning necessary actions supportive of their goal. (s)

Participant C, 20–25 y.o.

We don’t drink alcohol, don’t smoke. We exercise, drink more water than juices, eat fruit. Some things I haven’t changed yet, but I’d really start to prepare for pregnancy at least three months before we plan to start trying for a baby. Three months to a year, actually.

Participant I, 20–25 y.o., mother of 1, 1 miscarriage

A lot is known about the effects of smoking, for yourself and the people around you, but especially for your unborn child. The risk it brings … Clubbed feet for example, lung conditions. That was something I could actively prevent by quitting, so I did.

### 2.4Unaware & competent

This section shows two quotes from participants that displayed behaviour in line with striving for their goal to having a healthy baby. This behaviour was present or altered prior to them being aware of having this goal.

Participant J, 20–25 y.o., mother of 2

I have always minded my nutrition, but only actively started thinking about it when I was pregnant. Maybe because I read something about it then, I’m not sure.

Participant H, <20 y.o., mother of 1, pregnant, 5 miscarriages

I quit energy drinks when I wanted to get pregnant. Well, no actually 6 months before that because I thought it might help relieve my endometriosis pains. I’ve had a lot of heart palpitations in the past as well and when the doctors couldn’t find anything wrong, I was relieved and wished to keep it that way. So I decided to try to quit energy drinks.

This figure provides a visual presentation of the two main themes; “The origin and development of personal goals” and “Awareness and competence”. For the second main theme, a visualization of the interrelationships is provided, detailing the process of becoming aware and competent with regard to one specific goal, as has been described by participants reflecting on their own (prior un)awareness and (in)competence.

Awareness refers to the level of awareness a participant has with regard to a specific goal they might wish to achieve, or the awareness that actively striving for this goal is a realistic possibility for them. Competence refers to actively performing behaviour that aligns with striving for a particular goal. In this study, (in)competence is not a judgement of moral character, but an evaluation of participants’ current behaviour in relation to their goal.

## Discussion

In this study, we have explored the concept of maternal health agency in women with a low SES. The secondary aim was to explore if the conceptualization of health agency as presented in Figure 1 is suitable to better understand the low uptake of PCC in this population. A summary of the interpretation of the main results can be found in Appendix C.

### Interpretation of the results

Two main themes were identified; 1. The origin and development of goals and 2. Awareness and competence. The first main theme was focused mostly on identification of main goals (such as having a healthy, happy child) and the sub goals that were supportive of achieving the main goals (such as the child having friends). The second main theme focused on participants’ practical ability to achieve their (main and sub) goals. This entails their past and current level of awareness and competence regarding their goals, the implications of the choices they made, and their reflection on said choices.

#### Sources of goal-awareness

Incitement of goal-awareness came from three main sources; cultural normativity, personal narrative and intuition. Participant B, who said that her *only* goal is for her child to be healthy, demonstrates how these sources are not mutually exclusive. Having a healthy baby is considered a normative goal, as society expects mothers to wish for a healthy child (Pedersen, 2016). When asked why this was her *only* goal however, participant B explains that a child in her family suffers from a heart condition, which impacted how she values health. This witnessed experience became part of her personal narrative, thereby inciting or strengthening her awareness regarding her goal.

Knowing or feeling what to do intuitively was identified as another source of goal-awareness. As a parenting theory, intuitive parenting refers to prioritizing connection and cooperation over fear-based discipline. In a biological sense however, it refers to instinctively knowing what to do (Papoušek & Papoušek, 2002) and how to do it right (Wilkins, 2006), such as the natural inclination (in physiological circumstances) to sooth a crying infant. When soothing is ineffective, the parent becomes aware of a possible deficit in their knowledge and their (perceived) incompetence, prompting further action. In other words, parental intuition acts either as a guide to “do the right thing” or as a stimulus to actively search for “the right thing”. It must be kept in mind that a strict distinction between normative, narrative and intuitive origins of goals can neither be upheld in research nor in practice, because intuition and personal narrative may be shaped by what is deemed normal, and what is deemed normal may be shaped by intuition and narrative.

#### Attribution of value to goals

A distinction was noticed between goals that were listed and personal goals that were mentioned solitary. Personal goals were often spontaneously accompanied by the underlying reasons for the goal, implying that the participant became aware of this goal and attributed high personal value to it, at a time prior to the interview. The manner in which listed goals were mentioned, as if from a checklist, suggests that participants perceived them as belonging to a certain theme. This is in accordance with what is known about the cultural normativity of motherhood, which is heavily influenced by the media and dictates which goals “*a good mother*” should strive for (Douglas & Michaels, 2005). It is apparent that certain listed goals were personally valued highly by participants as well, as some of these goals were elaborated on at a later time in the interview. This indicates that, apart from the goal being valued highly by society, the participant also had a personal reason to value this goal.

#### Balancing benefits and burdens

Logically, the first step of actively (and therefore consciously) striving for a goal is to be aware of it. In our data, we found that becoming aware of a goal led to the participants attributing personal value to this goal, meaning that they considered if this goal was something they would wish to achieve, and if so, what cost would be acceptable. When high attributed value was combined with low associated burdens, the goal became integrated in their life, meaning they actively strived (or planned to strive) for it. When the burdens outweighed the benefits, participants either showed they became overwhelmed, or adapted their goal to fit the burdens they could and were willing to bear. This can be interpreted as balancing out the benefits and burdens in order to reach an equilibrium and avoid internal conflict caused by a discrepancy between one’s goal and one’s behaviour.

#### Adequate maternal health agency

Proper balancing requires sufficient comprehension of the consequences associated with either choice: striving of not striving for a goal. How these consequences are viewed however, is influenced by multiple factors such as preferences, risk assessment, current level of (in)competence, and the (perceived) likelihood of success. Balancing therefore, should be viewed as a dynamic process, of which the outcome can sometimes be odds with normative goals such as “good mothers” indefinitely prioritizing their children’s needs (Douglas & Michaels, 2005; Wilkins, 2006). In other words, adequate health agency does not necessarily lead to choosing the objectively most beneficial option, but rather leads to being aware and competent, striving to achieve a personally valued goal at a cost one is able and willing to pay.

### A concept of health agency

The secondary aim of this study was to explore if the conceptualization of health agency (Figure 1) is suitable to better understand the low uptake of PCC in this population. The three pillars seem to offer guidance to check if the basic conditions needed for adequate health agency are met, which was the case for all participants in this study, as all were able to form goals, felt at least moderately in control of achieving these goals, and indicated that their freedoms and means were sufficient, or that they had reasonable ways to make it so. However, examples of possibly diminished health agency were present in this study, despite our findings on meeting the three pillars of health agency.

#### Diminished health agency

In this study, causes of diminished health agency resulted from, for instance, a goal not (yet) being integrated, rejection or overwhelmedness, or the participant not perceiving the relevance of striving for their goal at that particular time (i.e., unawareness). This last example is demonstrated by participant E who underwent fertility treatments and described how she worried about having an eating disorder and having a baby. She did not discuss this with her fertility specialist because, at the time, she did not deem the topic relevant. When participant E became pregnant, the topic became relevant to her and she initiated a conversation with her midwife on dietary habits. Participant E met the three pillars of health agency (Figure 1) as she was able to form her goal to have a healthy baby, felt in control with regard to her ability to discuss the eating disorder with a healthcare provider, and had the possibility (means) to discuss this topic during a planned consultation. Despite meeting these pillars however, participant E experienced diminished health agency due to a knowledge deficit regarding the impact of dietary habits during fertility treatment (Twigt et al., 2012). In other words, unawareness prevented her from actively striving to have a healthy baby during the preconception period.

#### Understanding the uptake of PCC

These findings indicate that the low uptake of PCC cannot be better understood through the three pillars of health agency as they are currently described, and is more likely explained by a lack of awareness of the usefulness of PCC. Incitement of goal-awareness however, could be viewed as a part of the first pillar (“The capacity to form health-goals one has reason to value”), as unawareness negatively affects one’s capacity to form certain goals.

Our findings with regard to awareness of the necessity and usefulness of PCC corroborates the findings of Maas et al. regarding the difference between planning a pregnancy and adequately preparing for it (Maas, Poels, de Kievit, et al., 2022). Most participants in their study, as well as in this one, did not perceive striving to have a healthy baby as a relevant goal during the preconception period. It seems clear therefore, that the first step towards supporting maternal health agency in women with a low SES, as well as the general population, lies in aiming to resolve the knowledge deficit and boost awareness of the relevance of striving for a healthy baby during the preconception period.

### Implications for practice

Addressing inequity in adverse pregnancy outcomes between groups with a different SES is a complex and challenging matter. Apart from medical interventions and social policies, interventions should be focused on inciting goal-awareness and supporting goal-integration in order to increase the demand for PCC and improve its uptake in women with a low SES. The importance of goal-awareness is supported by examples we encountered in our data, in which participants stated that they wished they had acted differently in the past, had they known that their actions affected their goals. These findings are in alignment with the findings of Ismaili M’hamdi et al. (M’hamdi et al., 2018) and Poels et al. (Poels et al., 2016) who have reported that women wish for a healthy baby but often are not aware of, or fully believe in, their own influence regarding the outcome of their pregnancy.

Apart from inciting goal-awareness, guidance and empowerment through education on how to take action is paramount. Scaffolding women’s own inclination to actively strive for their goals will lead to a more durable change than imposing healthcare when and where we, as healthcare professionals, deem necessary. A possible way of stimulating goal-awareness as well as education lies in normalizing open conversation on pregnancy related topics between prospective parents and their peers, as well as between healthcare providers and their patients. For example, television commercials and other forms of advertising could be used to promote knowledge of the preconception period and PCC, as is often done for other government funded health promotion such as smoking cessation campaigns. Similar actions were researched by Maas et al., who conducted a study on local facilitation of goal-awareness incitement regarding PCC through developing the social marketing strategy Woke Women® (Maas, Blanchette, et al., 2022). This strategy was aimed at empowering women to actively prepare for pregnancy in 6 municipalities in the Netherlands and showed tentative positive effects on lifestyle behaviours among prospective parents (Maas, Poels, Ista, et al., 2022).

### Strengths and limitations

Public health policies are usually aimed at improving availability and accessibility of health care. Studies on health literacy and health care uptake however, show that the demand for care plays an important role in the actual uptake (Lee et al., 2021; Tschaftary et al., 2018). This implies that effective, high quality health care should include supporting the demand for care within its target group. Our findings support this and provide a different perspective on ways to address the high incidence of adverse pregnancy outcomes in women with a low SES. Furthermore, to the best of our knowledge, this study is the first to research maternal health agency in women with a low SES by involving the target group themselves. This provides clear and tailored clues, suggesting that further research should be aimed at exploring the effects of inciting goal-awareness to support and empower these women.

A possible limitation of this study lies in selection bias, as it is known that women who apply for interviews may have a higher natural inclination to act than those who do not participate. Therefore, the women that have participated in this study might not belong to the group whose health agency is most impeded by their social circumstances. We have chosen to accept this limitation because the women we have interviewed still belong to a group with a high incidence of adverse pregnancy outcomes which warrants action to reduce these outcomes.

For future research, we suggest that women with a high- and intermediate SES are included as well, in order to evaluate differences and similarities between both groups.

## Conclusion

In this study, we aimed to explore the concept of maternal health agency and discover which factors influence health agency in women with a low SES. The secondary aim was to explore if the conceptualization of health agency as presented in Figure 1 is suitable to better understand the low uptake of PCC in this population.

The results of this study indicate that maternal health agency cannot be viewed as a static personal or population trait, but rather should be viewed as a changeable outcome that hinges on goal-awareness and is dependent on multiple changeable factors, specific to a person, time, place and goal. All participants seemed to meet the three pillars of health agency (Figure 1), but examples of diminished health agency were also found. The missing link between meeting the pillars and having adequate maternal health agency seemed to be a lack of awareness with regard to the relevance of a certain goal at a specific time, which diminishes the ability to take action and strive to achieve it.

It must be taken into consideration that the high incidence of adverse pregnancy outcomes in women with a low SES is rooted in their social circumstances, and therefore attempts to improve these circumstances should always be made. In addition to these attempts, supporting and strengthening maternal health agency in these women may also have a lasting and positive effect on their pregnancy outcomes. A possible first step to do so, is to provide them with stimuli that can incite goal-awareness regarding having a healthy baby, or more specifically; to incite awareness of which (sub) goals they can actively and realistically strive for to help them have a healthy baby.

## Data Availability

The dataset of this study is available from the corresponding author on reasonable request.

## Abbreviations

Preconception Care: PCC
Developmental Origins of Health and Disease: DOHaD
Socioeconomic status: SES

## Appendix A

### An overview of additional participant characteristics

**Table:**

Participant	Age	Obstetric background	Maternal status
A	20–25		Plans to become pregnant within 3 years
B	25–30	>20 weeks pregnant	Pregnant with her first child
C	20–25		Plans to become pregnant within 3 years
D	25–30		Mother of 5 children, 0–5 years of age
E	30–35		Mother of 1 child, 2 months of age
F	25–30		Mother of 1 child, 7 months of age
G	20–25		Mother of 1 child, 2 months of age
H	<20	5 miscarriages >20 weeks pregnant	Mother of 1 child, 18 months of age
I	20–25		Mother of 1 child, 8 months of age
J	20–25		Mother of 2 children, 2 months & 2 years of age
K	30–35	Actively trying to have a baby for over 3 years	Stepmother of 1 child, 8 years of age
L	35–40	2 miscarriages	Mother of 2 children, 2 & 8 years of age
M	30–35		Plans to become pregnant within 3 years
N	30–35	2 miscarriages	Mother of 4 children, 0–13 years of age
O	35–40	1 abortion 1 miscarriage	Unprotected intercourse, but not trying to have a baby

## Appendix B

### The topic list

#### Grand tour

Participant without children So you don’t have any children at this moment, but you stated to wish to have a child in the future. Could you elaborate a bit on that wish?

Participant who is a mother → You filled in that you have a child/children. Could you please tell me something about your family?

#### Interview questions

The capacity to form health-goals one has reason to value

1. Women who wish to have a baby usually focus most on becoming pregnant. Which goals do you have/did you have in addition to this prior to pregnancy?
  - When did these goals first arise?
  - What was it triggered by?
  - Was there a specific moment you started to act on having a healthy baby?
  - What did you do?
  - Could you find what you were looking for?
2. What comes to mind when you think about the wellbeing of your (future) child?

a. Was this on your mind prior to pregnancy?

- If not, when di dit start? What was the trigger?
- From which moment onwards do you think you can affect your (future) child’s wellbeing and health?
- And in what way?
- Has this changed in any way? If so; howcome?

3. What does ideal motherhood look like to you, with regard to how you vision it and which goals you(‘d) have?

- How did you develop this view/these goals?
- Why are these important to you?
- Did these goals change at any time? If so, howcome?

The control one perceives to have over achieving those health-goals

4. How would you go about to achieve your goals?

- If applicable; when did you plan this out?

5. Did you look for support or information?

- If so, were you able to find what you needed?
- If not, was there a specific reason you did not?

6. How do you feel about achieving your goals?

- Do you see any barriers?
- Do you see ways to overcome possible barriers?

7. Do you feel that the outcome of pregnancy can be influenced?

- Has this view ever changed?
- If so, why?

The freedom(s) one has to achieve those health-goals

Do you feel you have sufficient means (time, support, motivation, space, money etc.) to achieve the goals you have mentioned? c. If so, can you elaborate? d. If not, can you elaborate?

- Why is … insufficient?
- Do you see a way of resolving this insufficiency?

8. Has your view on the sufficiency of your means changed at any moment?

Ending

9. Are there things we have not discussed which you deem important or would like to share?

## Appendix C

A structured summary of the interpretation of the main results

Main themes

The origin and development of goals:

- Main goals (e.g., having a healthy, happy child) and supportive sub-goals (e.g., the child having friends).

Awareness and competence:

- Awareness refers to one’s awareness of (the realistic achievability of) goals
- Competence refers to one’s ability to achieve a goal and choosing to actively strive for it
- Competence does imply judgement of moral character

### Sources of Goal-Awareness

1. Cultural normativity:

Societal norms, values and expectations (e.g., mothers always wish for a healthy child)

2. Personal narrative:

Personal experiences impacting goal valuation (e.g., having a family member with a medical condition)

3. Intuition:

Instinctive knowledge and actions in parenting (e.g., soothing a crying infant)

### Attribution of Value to Goals

- Personal goals are often spontaneously elaborated on
- Listed goals seem to belong to a category, based on societal norms and values.
- Listed goals can also be personally valued highly

### Balancing Benefits and Burdens

- Awareness of a goal leads to attribution of personal value
- The value attributed to (the benefits of) achieving a goal are compared to the expected burdens associated with striving for it
- The outcome of balancing benefits and burdens is influenced by probability as well as personal characteristics such as risk perception.

### Adequate Maternal Health Agency

- Adequate agency requires sufficient understanding of the consequences associated with striving or not striving for a goal
- Adequate health agency does not necessarily mean objectively making the most beneficial choice
- Entails being able to strive for goals one personally values, at a cost one is able and willing to bear

### A Concept of Health Agency

Diminished Health Agency

- Despite all participants meeting the three pillars of health agency (Figure 1), diminished health agency was encountered
- Causes of diminished agency included unintegrated goals, rejection and overwhelmedness and perceived irrelevance of a goal at a certain time

Understanding the Uptake of PCC

- The low uptake of PCC cannot fully explained by the three pillars of health agency.
- Lack of awareness regarding the usefulness of PCC is more likely the cause of its low uptake
- Resolving the widespread knowledge deficit on the usefulness of PCC through boosting awareness is crucial for supporting maternal health agency in all populations
